# Mitigating Doxorubicin-Induced Skeletal Muscle Toxicity: A Review of Oxidative Stress Mechanisms and the Therapeutic Role of Exercise

**DOI:** 10.3390/antiox14070870

**Published:** 2025-07-16

**Authors:** Quinten W. Pigg, Dillon R. Harris, Daniela Sayuri Inoue, Mariana Janini Gomes

**Affiliations:** Department of Kinesiology and Sport Management, Texas A&M University, College Station, TX 77845, USA; qwpigg99@tamu.edu (Q.W.P.); dillon_harris@tamu.edu (D.R.H.); dani.yoshimura_7@tamu.edu (D.S.I.)

**Keywords:** chemotherapy, reactive oxygen species, metabolism, resistance training, endurance training

## Abstract

Doxorubicin (DOX) is a highly effective chemotherapy drug used in the treatment of many cancers, including solid tumors, hematological malignancies, and soft tissue sarcomas. Despite its potent antitumor effects, DOX is known to have toxic effects in non-tumorous tissues, such as skeletal muscle. Potential mediators of DOX-induced skeletal muscle toxicity are reactive oxygen species (ROS). An overproduction of ROS can disrupt the balance between oxidants and antioxidants in a cell, leading to oxidative stress. Chronic oxidative stress has been shown to upregulate proteolysis, ultimately leading to muscle wasting. Exercise stands as a potent nonpharmacological therapy capable of attenuating muscle wasting by enhancing metabolic function and antioxidant defenses while suppressing harmful ROS production. This review focuses on the current understanding of the role of oxidative stress in DOX-induced skeletal muscle toxicity. In addition, we highlight the effects of various exercise types on oxidative stress and muscle remodeling during DOX chemotherapy.

## 1. Introduction

Over 18 million cancer survivors were reported in the United States in 2022, with anticipated growth to 26 million by 2040 [1,2]. Advances in cancer screening and treatment have resulted in improved survivorship, but this improved survivorship does not necessarily equate to improvements in quality of life. Adverse consequences of cancer treatment linger or emerge years later, posing a threat to the quality of life and well-being of survivors over the long term [3].

Doxorubicin (DOX) is a highly effective chemotherapeutic agent used in the treatment of many cancers, including solid tumors, hematological malignancies, and soft tissue sarcomas [3]. Despite its potent antitumor effects, DOX is known to have toxic effects in non-tumorous tissues [4,5]. Cardiotoxicity is the leading adverse effect seen in cancer patients treated with DOX, which is managed by controlling the dosage patients receive to a maximum lifetime cumulative dose of 450–550 mg/m^2^ [6]. However, the adverse effects, even when carefully monitoring dosage, are not limited to cardiotoxicity. DOX causes skeletal muscle toxicity, resulting in wasting, fatigue, and weakness, which affect patients’ lives not only during therapy but for years following the cessation of therapy [7].

**Figure 1:**
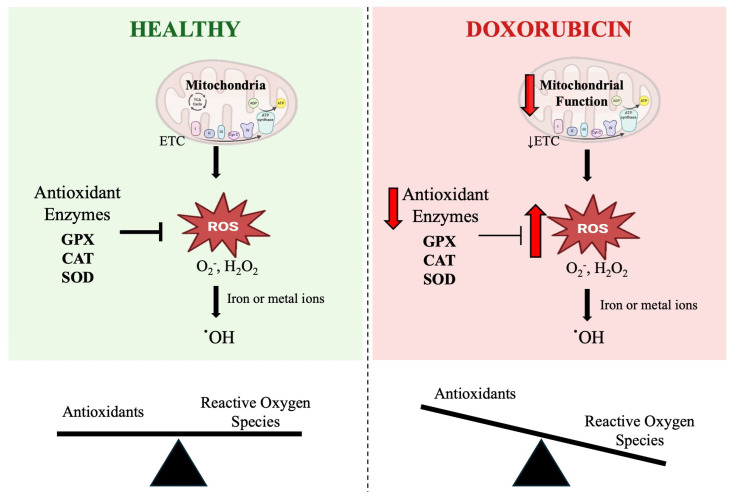
Schematic representation of the role of doxorubicin (DOX) in mitochondrial dysfunction-induced oxidative stress. The figure illustrates the differences in reactive oxygen species (ROS) regulation between healthy skeletal muscle and skeletal muscle exposed to DOX. Under healthy conditions (**left**), mitochondria produce ROS such as superoxide (O_2_^•−^) and hydrogen peroxide (H_2_O_2_) during respiration, which are detoxified by key antioxidant enzymes including superoxide dismutase (SOD), catalase (CAT), and glutathione peroxidase (GPX). This maintains a balance between ROS and antioxidants, preventing oxidative damage. In contrast, DOX treatment (**right**) impairs mitochondrial function and downregulates antioxidant enzyme activity, leading to excessive ROS accumulation. The presence of iron or metal ions further promotes the conversion of O_2_^•−^ and H_2_O_2_ to highly reactive hydroxyl radicals (·OH), which cannot be enzymatically neutralized. This imbalance between ROS and antioxidants results in oxidative stress and cellular damage. The scale below each panel reflects the shift from redox balance in healthy skeletal muscle to oxidative stress under DOX treatment. ETC: electron transport chain.

Potential mediators of DOX-induced skeletal muscle toxicity are reactive oxygen species (ROS) [8]. DOX can generate ROS through two mechanisms: disrupting mitochondrial respiration and through a nonenzymatic reaction with ferric iron (Figure 1) [9]. An overproduction of ROS can disrupt the balance between oxidants and antioxidants in a cell, leading to a condition known as oxidative stress. Oxidative stress can result in redox modifications of proteins, lipids, and DNA, negatively affecting both the cell and the whole organ function [10].

Despite the clinical implication of DOX-induced skeletal muscle toxicity, there are still no effective pharmacological therapies to protect skeletal muscle in this condition. Various treatments to manage muscle wasting have been explored, including nutrition and exercise interventions [11]. Exercise is known to have many benefits, including antioxidant and anti-inflammatory effects. Both endurance training (such as running or cycling) and resistance training (RT; such as weightlifting) have shown potential in protecting muscles from DOX damage by reducing catabolism, upregulating anabolism, and controlling inflammation and ROS production [12,13].

This review focuses on the current understanding of DOX-induced skeletal muscle dysfunction, specifically the role of oxidative stress. We also explore exercise as a strategy to protect skeletal muscle mass and function during DOX chemotherapy, highlighting the effects of various exercise types on oxidative stress and muscle remodeling.

## 2. Reactive Oxygen Species and Oxidative Stress

Oxidative stress is defined by an imbalance between ROS production and the body’s ability to neutralize them [14]. ROS, also known as free radicals, are essential for physiological cellular processes, playing important roles in redox signaling and cell survival, but chronic elevation in ROS levels has negative effects, such as oxidative damage to DNA, proteins, and lipids, as well as cell death [10]. The major sources of ROS include mitochondria, peroxisomes, endoplasmic reticulum, NADPH oxidases, and xanthine oxidase [15]. DOX is suggested to cause oxidative stress by inducing mitochondrial dysfunction, which ultimately results in the activation of proteolytic pathways.

In general, the primary source of ROS comes as a byproduct of mitochondrial respiration. Specifically, mitochondrial respiratory complexes I and III contribute mostly to the production of the free radical superoxide (O_2_^•−^) and hydrogen peroxide (H_2_O_2_), which, under normal physiological conditions, is rapidly detoxified via antioxidant molecules [16]. The skeletal muscle contains antioxidant systems that are responsible for neutralizing free radicals to maintain a healthy physiological level of ROS in the cell. The enzymatic antioxidant system is composed of the enzymes superoxide dismutase (SOD), catalase (CAT), and glutathione peroxidase (GPX), all of which play a role in neutralizing free radicals. Preclinical and clinical studies have shown that antioxidant activity is reduced in DOX treatment and positively associated with the beneficial effects of exercise [17,18].

Additionally, DOX can generate ROS through a direct interaction with iron or other metal ions. Both radicals O_2_^•−^ and H_2_O_2_, in the presence of iron and oxygen, can form a hydroxyl radical (•OH), which is highly reactive and does not have the ability to be enzymatically broken down, resulting in DNA damage [19]. The abundance of •OH, enhanced with DOX, serves to cause copious amounts of damage to mitochondrial DNA, the heart, skeletal muscle, and other systemic organs such as the liver and kidneys [20,21,22].

## 3. Role of Oxidative Stress in DOX-Induced Skeletal Muscle Wasting

DOX presents a paradox in the context of cancer-related muscle wasting. While it helps to eliminate cancer, potentially slowing and preventing further muscle loss caused by the disease itself, DOX is also known to independently induce skeletal muscle wasting. As a result, its use can ultimately worsen skeletal muscle loss, despite its anti-cancer benefits [23].

Maintenance of skeletal muscle mass depends on a delicate balance between protein synthesis and protein degradation. Skeletal muscle loss results from an increase in protein breakdown and/or a decrease in muscle protein synthesis. Muscle loss is a multifactorial and not completely understood condition that occurs in several systemic disorders [24]. Although the exact molecular mechanisms underlying DOX-induced muscle atrophy are not fully elucidated, the current literature supports the redox disturbances in skeletal muscle as a primary factor [25]. The increased production of ROS plays a central role in DOX-induced skeletal muscle wasting and dysfunction by promoting the activation of all major proteolytic systems (the ubiquitin–proteasome system, the autophagy–lysosomal system, and caspase-mediated apoptosis) [26].

**Figure 2:**
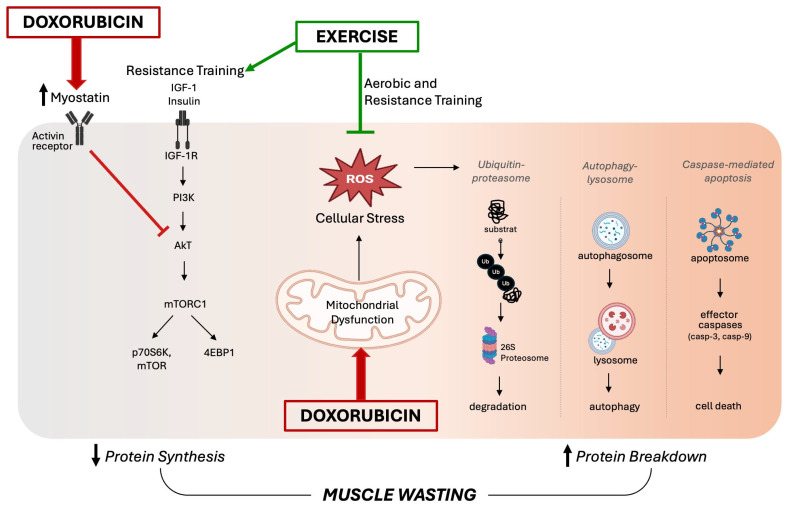
Proposed schematic diagram of intracellular signaling pathways involved in doxorubicin-induced skeletal muscle wasting. On the left side, disrupted insulin signaling by myostatin activity impairs the anabolic pathway, leading to reduced protein synthesis. In the center, doxorubicin-induced mitochondrial dysfunction results in an excessive accumulation of reactive oxygen species (ROS), triggering oxidative stress. This promotes the activation of major proteolytic systems—including the ubiquitin–proteasome system, the autophagy–lysosomal system, and caspase-mediated apoptosis—shown on the right. Together, these mechanisms drive skeletal muscle wasting. On the other hand, exercise counters these effects by reducing oxidative stress and limiting protein degradation. Additionally, resistance training stimulates mTOR signaling, supporting increased protein synthesis.

Specifically, the disruption of mitochondrial respiration due to DOX treatment leads to an excessive production of ROS, which triggers the activation of calpain and caspase systems, promoting protein degradation and muscle atrophy. The role of oxidative stress in the pathogenesis of muscle atrophy during DOX treatment has been evidenced by therapies targeting redox balance, such as supplementation with Vitamin C [27] and the antioxidant enzyme extracellular superoxide dismutase [18]. It has also been demonstrated that inhibition of autophagy prevented DOX-induced skeletal muscle atrophy and contractile dysfunction in female rats, and this protection was associated with an improvement in the oxidative stress profile [8]. Although several preclinical studies have shown that DOX-induced oxidative stress triggers muscle atrophy via the upregulation of proteolytic pathways, fewer studies have found a strong relationship between DOX administration and reduced protein synthesis [25]. A potential mechanism for DOX-induced muscle wasting involves reduced protein synthesis mediated by myostatin, which is upregulated following DOX treatment [28]. In addition to inhibiting protein synthesis, myostatin can promote muscle protein degradation by activating FOXO transcription factors, which in turn increases the expression of atrophy-related genes such as atrogin-1/MaFbx and MuRF-1 [29]. Additionally, DOX treatment can disrupt insulin signaling by reducing the expression of critical proteins such as GLUT4 and AMPK that are involved in glucose uptake, which ultimately impairs protein synthesis [25]. Figure 2 summarizes the key intracellular signaling pathways involved in DOX-induced skeletal muscle wasting, as discussed in this section.

## 4. Role of Exercise Against DOX-Induced Skeletal Muscle Wasting

Exercise is well known for its multi-systemic protective benefits during several pathological conditions [30,31,32], and increasing evidence from preclinical and clinical studies suggests that exercise can provide protection to multiple tissues affected by DOX chemotherapy [21,33,34]. Furthermore, exercise preconditioning may attenuate DOX-induced cytotoxicity in both cardiac and skeletal muscles [12,34,35]. Both anabolic and catabolic muscle pathways are strongly influenced by physical exercise [36]. However, the mechanisms underlying the protective effects of exercise on muscles with regard to DOX have not yet been fully elucidated. This section discusses the current understanding of the effects of exercise training on DOX-induced skeletal muscle wasting and dysfunction, with a focus on the role of oxidative stress.

It has been well established that exercise has antioxidant and anti-inflammatory properties because it affects many redox-sensitive signal transduction pathways [37]. In addition, exercise training has anti-apoptotic effects through modulation of the caspase-dependent apoptotic pathway [38]. In the context of DOX-induced skeletal muscle toxicity, it has been suggested that exercise provides protective effects against DOX-induced oxidative stress in skeletal muscle by increasing endogenous antioxidants, improving mitochondrial biogenesis and oxidative capacity, modulating important markers of apoptosis and autophagy, and reducing inflammatory activation pathways [12,39,40]. 

The previous literature shows that exercise enhances antioxidant activities in cardiac and skeletal muscle by upregulating various cellular antioxidant enzymes such as SOD, CAT, and GPX [13,41]. However, most of the investigations into the protective effect of exercise against DOX-related adverse effects have focused on the cardiac muscle while the effects of exercise on DOX-induced skeletal myopathy have been less explored.

Preclinical investigations of the acute effects of DOX often involve a single injection at a dose of 20 mg/kg in rodent models. Studies have shown that DOX administration at this dosage leads to acute negative effects in skeletal muscle, including increased stress, protease activation (calpain and caspase-3), and autophagy-related genes (*Beclin-1*, *ATG12*, *ATG7*, *ATG12-ATG5*, and *LC3*) [8,42]. However, aerobic preconditioning can prevent these negative effects, which is suggested to happen due to an increase in the muscle levels of antioxidant enzymes and heat shock proteins (HSPs) [43]. Specifically, HSP72 may be crucial to cardiac and skeletal muscle health in the context of DOX treatment. HSP72 is highly regulated by exercise and plays a role in reducing cardiotoxicity and myotoxicity by upregulating and shuttling antioxidant defenses such as SOD and GPX in the mitochondria [42]. As previously mentioned, SOD and GPX are enzymes known for their role in neutralizing free radicals upregulated by DOX [17,18]. Enhancing these factors produced by exercise, also referred to as exerkines, serves to promote a protective effect against DOX-induced oxidative stress, thus potentially ameliorating myotoxicity [44,45]. Recently, there has been a growing interest in exerkines, and more studies are needed to elucidate their importance in the context of DOX. However, a comprehensive review on the role of exerkines in modulating exercise adaptations involved with redox signaling in health and disease can be found here [46].

The pro-oxidant effect of DOX treatment persists in the long term as well, evidenced by an increase in lipid peroxidation and protein carbonylation (markers of oxidative damage to lipid and proteins, respectively), and a decreased antioxidant activity [47,48]. Chronic administration of DOX disrupts both oxidative metabolism and autophagy in skeletal muscle [8,42,47]. The downregulation of mitochondrial complex activities impairs energy production, while the dysregulation of autophagy leads to an imbalance in protein turnover. These two factors together result in the progressive degradation of muscle tissue, ultimately contributing to skeletal muscle wasting [40,47,49]. In contrast, long-term aerobic training has been shown to improve autophagy function and repair myogenic differentiation in mice chronically treated with DOX [40]. Both preconditioned aerobic training and post-DOX treatment aerobic training cause improvements in oxidative stress and inflammatory markers when compared to DOX sedentary counterparts. However, the combination of preconditioning and post-exercise therapies was the most effective nonpharmacological strategy for minimizing the harmful long-term effects of DOX in skeletal muscle [50].

Most of the current literature focuses on the effects of aerobic training before and during DOX treatment, while less is known about the impact of RT on DOX-induced skeletal muscle dysfunction. Exercise-induced adaptations in skeletal muscle are specific to the type of training: aerobic training primarily promotes metabolic and mitochondrial adaptations, enhancing endurance capacity, while RT more directly stimulates muscle hypertrophy and strength gains [51]. One key mechanism that facilitates muscle growth is the mechanistic target of rapamycin complex 1 (mTORC1), which regulates essential processes such as protein synthesis and autophagy [52,53]. Bodine (2022) described a comprehensive review of the mTOR pathway’s regulation [53]. In brief, repeated increases in mechanical loading as a result of RT activate insulin-like growth factor 1 (IGF-1), which triggers a signaling cascade involving phosphoinositide 3-kinase (PI3K) and protein kinase B (AKT). This leads to the phosphorylation of the tuberous sclerosis complex-1/2 (TSC-1/2) and activation of the mTORC1 pathway, resulting in either muscle growth and repair (via mTOR and S6K1) or autophagy (via 4EBP1) [53].

Studies on skeletal muscle response to RT in subjects treated with DOX are scarce. Bredahl et al. conducted a series of investigations using a rat model of low-intensity RT; the model progressively loaded the rats’ hindlimbs by employing a raised cage to reach food and water. This RT protocol was shown to mitigate the decline in skeletal muscle function induced by DOX in the rats [34,54,55]. However, the molecular mechanisms underlying these effects were not explored and remain to be elucidated. Currently, there is a lack of clinically relevant studies conducted with RT in both humans and animals associated with cancer and cancer treatment. The current guidelines for RT during and after cancer treatment are undeveloped due to a lack of research and therefore generally mirror standard health guidelines [56]. There is a growing need to understand how RT may be an effective strategy to mitigate DOX-related muscle dysfunction. Although the current literature is limited on DOX and RT, this gap presents numerous opportunities for researchers to explore potentially beneficial mechanisms by which RT may mitigate DOX-induced oxidative stress and skeletal muscle toxicity.

The processes of exercise-induced hypertrophy and chemotherapy-induced muscle wasting are distinct, but they do interact with common elements of the IGF1/PI3K/Akt signaling pathway [25]. As previously mentioned, DOX increases ROS generation, which inhibits IGF1 signaling [25]. In contrast, RT stimulates the IGF1 pathway, leading to mTORC1 activation [57]. Given the physiological response of skeletal muscle to exercise, it is plausible that the preservation of skeletal muscle mass is possible via RT and may attenuate the degree of DOX-induced muscle wasting. However, skeletal muscle under DOX treatment has shown increased oxidative stress and inflammation, reduced protein synthesis, and an impaired response to anabolic stimuli (termed anabolic resistance) as a result of DOX treatment [25,27,50]. Therefore, further research is necessary to understand how a muscle’s response to different types of exercise is affected by DOX exposure. Tailored exercise regimens, considering factors such as duration, frequency, intensity, and type, are essential to maximize efficacy in mitigating muscle wasting. Further investigations combining various exercise types with nutritional interventions are needed to identify effective strategies for improving muscle mass during DOX treatment.

## 5. Future Directions

Despite growing evidence supporting exercise as a countermeasure to DOX-induced skeletal muscle toxicity, several important research gaps remain. Traditionally, most research has emphasized DOX-induced cardiotoxicity and the use of endurance training to mitigate these effects. While important, this focus has led to a gap in understanding the equally critical issue of skeletal muscle wasting and dysfunction, which significantly impacts survivors’ quality of life and physical independence. Similarly, most studies have focused on endurance-based training, whereas the role of resistance training—alone or in combination with endurance modalities—remains underexplored in both preclinical and clinical settings.

Future studies should investigate a range of exercise models, including resistance training, concurrent endurance and resistance training, and interval training, to identify the most effective strategies for preventing muscle atrophy while also supporting cardiovascular health in the context of DOX treatment. Moreover, mechanistic studies are also needed to better understand how different exercise modalities influence mitochondrial function, oxidative stress, and muscle protein turnover under both acute and chronic DOX exposure, as well as the potential role of exerkines in modulating these processes. Finally, longitudinal research is essential to assess the long-term recovery of skeletal muscle following the cessation of DOX chemotherapy. Addressing these gaps will be critical for developing personalized, effective exercise interventions that improve survivorship outcomes.

## 6. Conclusions

DOX-induced skeletal muscle toxicity involves complex mechanisms, including mitochondrial dysfunction, oxidative stress, and skeletal muscle wasting. Exercise has shown promise as an effective intervention to mitigate these adverse effects, improving muscle health and patient outcomes. However, critical gaps remain—particularly regarding the role of RT and the underlying protective mechanisms of exercise. Furthermore, understanding the long-term recovery of skeletal muscle after DOX treatment is essential. Addressing these gaps will be key for refining exercise interventions and enhancing the quality of life and survivorship of cancer patients undergoing DOX chemotherapy.
